# Deep underground metagenome-assembled genomes from hydrothermal spring

**DOI:** 10.1128/mra.00574-24

**Published:** 2024-09-16

**Authors:** Kirill Tarasov, Elena Kravchenko, Mikhail Zarubin, Alena Yakhnenko

**Affiliations:** 1 Joint Institute for Nuclear Research, Dubna, Russia; Montana State University, Bozeman, Montana, USA

**Keywords:** hydrothermal spring, metagenome, deep underground, Elbrus, ngs

## Abstract

We report 19 metagenome-assembled genomes from a deep underground microbial community found in mineralized hydrothermal spring in the Baksan Neutrino Observatory tunnel. The community is predominantly occupied by members of Pseudomonadota (Gamma-, Beta-, and Alphaproteobacteria), Planctomycetota, Myxococcota, Nitrospirota, Cyanobacteria, Gemmatimonadota, and Armatimonadota.

## ANNOUNCEMENT

Deep underground microbial communities are of great interest to scientists but remain poorly studied due to their inaccessibility (1, 2). The Baksan Neutrino Observatory of the Institute for Nuclear Research of the Russian Academy of Sciences (Baksan Neutrino Observatory) is a unique scientific facility that allows for the study of microbial communities in deep underground mineral springs.

A biofilm sample from a mineral spring of the far unused part of the BNO tunnel (43°16′32.5″N, 42°41′30.3″E) (3) was collected in October 2020. The sample was frozen at −20°C and transported to the laboratory where it was stored at −80°C. A detailed description of the sampling area and mineral source is given in Tarasov et al. (4). Four DNA extractions were performed from the biofilm sample using GeneJET Genomic DNA Purification Kit (Thermo Scientific). Each extraction yielded 0.9–1.5 µg of DNA that was sequenced separately; 200 ng of DNA was taken for sequencing. The four datasets were combined and analyzed jointly. Libraries were prepared from purified DNA using Hyper Library, Kapa Biosystems (Roche); their quality and quantity were assessed using the Fragment Analyzer and by qPCR. Sequencing was performed on an Illumina MiSeq sequencer (TruSeq v.3 kit). FASTQ files contained 31,061,948 paired-end reads 300 bp long, which were obtained using bcl2fastq (v.2.20 Conversion Software, Illumina). All software was used with default parameters unless otherwise noted. Trimming and processing of reads were performed using HTStream (v.1.3.2) (5), where hts_QWindowTrim was run with a width parameter (-w) of 10 and a quality cutoff parameter (-q) of 20. Quality control was assessed before and after processing with FastQC (v.0.12.1) (6) and MultiQC (v.1.14) (7). Deduplicated reads without adapter sequences and with *Q*-scores of >20 were assembled using MetaSPAdes (v.3.15.5) (8) with lengths of k-mers of 21, 33, 55, 77, 99, and 127. Contigs with lengths of >2,500 bp were assigned to bins in MetaBAT2 (v.2.12.1) (9). BWA mem 0.7.17-r1188 (10) was used for read mapping. Completeness and contamination of bins were assessed using CheckM (v.1.2.1) (11). Bin classification was performed using GTDB-Tk (v.2) (12) based on Genome Taxonomy Database release 207v2 (13). Raw reads were used to retrieve the 16/18S rRNA gene sequences using RiboTaxa (v.1.5) (14) with the NUM_ITERATION parameter of 50, as the database of SSU sequences SILVA (v.138) was used.

A total of 97.7% of raw reads successfully passed the trimming and quality control filters. Out of the 42 initially constructed bins, 19 that passed quality control quality grade “high” or “medium” according to classification by Bowers et al. (15) and completeness of ≥90%] were deposited to GenBank National Center for Biotechnology Information (Table 1). These 19 bins (metagenome-assembled genome) had a mean estimated coverage of 97× (with a minimum value of 11× and a maximum value of 856×) and accounted for 81.53% of the community. Bins with 16S rRNA gene present constituted 78.86% of the community .

**TABLE 1:** Accession numbers and assembly statistics of metagenome-assembled genomes from deep underground hydrothermal spring^a^

BIN number	Accession no.	Length	Reads	Completeness	Contamination (%)	TRNA	Contigs	Coverage (*x*)	N50	Phylum	GTDB taxonomy
Bin_1	JBDIVK000000000	2,888,395	5,368,687	97.13	0.62	20	58	445	80,241	Pseudomonadota	g__Nitrosomonas
Bin_2	–	3,171,675	233,564	69.57	24.86	15	672	9	4,995	Desulfobacterota	f__UBA2774
Bin_3	–	860,745	186,219	33.92	0	11	243	8	3,437	Pseudomonadota	g__PHEG01
Bin_4	–	409,113	23,984	0	0	0	25	12	25,653	Unclassified	–
Bin_5	JBDIVL000000000	3,907,353	331,075	98.9	2.75	19	100	19	70,499	Planctomycetota	f__Brocadiaceae
Bin_6	JBDIVM000000000	3,853,294	1,805,663	99.5	0	19	18	88	327,066	Pseudomonadota	g__Limibaculum
Bin_7	–	2,879,337	453,841	78.41	2.27	20	414	14	8,565	Planctomycetota	f__UBA1924
Bin_8	–	2,395,722	373,120	42.72	1.3	13	585	8	4,175	Pseudomonadota	g__SBBV01
Bin_9	–	371,619	46,103	1.37	0	1	64	11	6,535	Unclassified	–
Bin_10	–	2,352,692	264,372	50.95	2.84	14	607	8	3,487	Planctomycetota	__UBA1845
Bin_11	–	2,842,496	682,920	67.55	5.17	12	611	11	4,923	Pseudomonadota	f__JAHJQQ01
Bin_12	-	243,718	111,755	0	0	–	5	98	184,403	Unclassified	–
Bin_13	-	1,711,117	267,465	43.11	0.16	12	96	15	25,110	Pseudomonadota	g__Methylocella
Bin_14	JBDIVN000000000	3,490,454	375,291	96.58	1.28	20	23	17	207,837	Cyanobacteria	f__Vampirovibrionaceae
Bin_15	JBDIVO000000000	4,969,468	707,759	96.04	4.37	19	511	18	12,908	Pseudomonadota	s__Methylocaldum sp002005105
Bin_16	JBDIVP000000000	3,724,183	275,398	96.74	3.18	17	417	11	12,115	Nitrospirota	f__UBA8639
**Bin_17**	**JBDIVQ000000000**	**4,059,307**	**1,248,965**	**94.32**	**1.14**	**19**	**258**	**53**	**23,890**	**Planctomycetota**	**g__UBA5793**
**Bin_18**	**JBDIVR000000000**	**4,587,371**	**742,227**	**95.91**	**1.94**	**20**	**93**	**19**	**111,591**	**Myxococcota**	**g__CAADGG01**
**Bin_19**	**JBDIVS000000000**	**2,629,255**	**9,385,427**	**97.1**	**0.36**	**19**	**35**	**856**	**100,535**	**Pseudomonadota**	**f__Wenzhouxiangellaceae**
Bin_20	JBDIVT000000000	2,468,623	317,833	93.21	2.78	20	18	21	271,362	Armatimonadota	s__J051 sp003695835
Bin_21	–	3,071,481	277,371	80.61	7.12	14	572	8	5,998	Nitrospirota	f__Nitrospiraceae
**Bin_22**	**JBDIVU000000000**	**2,848,031**	**1,762,563**	**98.86**	**0**	**20**	**12**	**120**	**274,431**	**Planctomycetota**	**g__J020**
Bin_23	–	1,162,359	69,383	23.82	0	13	329	6	3,464	Acidobacteriota	g__OLB17
Bin_24	JBDIVV000000000	3,255,097	187,089	94.74	3.7	17	313	11	14,682	Pseudomonadota	s__Tatlockia maceachernii
Bin_25	JBDIVW000000000	4,512,728	633,322	96.76	5.45	18	77	27	98,413	Nitrospirota	f__Nitrospiraceae
Bin_26	–	4,058,730	404,999	66.65	2.64	15	753	8	5,952	Planctomycetota	f__Phycisphaeraceae
Bin_27	–	528,773	103,119	19.75	0	5	152	7	3,341	Pseudomonadota	__UBA828
Bin_28	–	2,150,631	542,064	73.09	5.05	13	405	11	6,050	Pseudomonadota	s__UBA2250 sp004323595
Bin_29	JBDIVX000000000	8,233,326	815,134	97.17	7.9	20	364	14	38,883	Myxococcota	f__SG8-38
**Bin_30**	**JBDIVY000000000**	**3,268,211**	**699,358**	**100**	**3.51**	**20**	**26**	**23**	**266,143**	**Planctomycetota**	**g__CAADGN01**
Bin_31	–	2,430,011	634,158	77.93	10.34	15	230	16	15,663	Pseudomonadota	g__Thiobacillus
Bin_32	–	12,765,779	1,300,226	99.84	218.56	–	2,289	9	6,236	Unclassified	–
Bin_33	–	3,484,374	769,983	85.74	7.88	15	545	10	7,566	Pseudomonadota	g__Pelomicrobium
Bin_34	–	2,747,306	93,967	41.38	0	12	671	7	4,108	Chloroflexota	g__JADJUV01
Bin_35	–	6,747,043	548,927	82.19	2.33	19	835	10	10,289	Planctomycetota	f__H5-PLA8
Bin_36	–	599,647	60,967	0	0	–	63	13	12,907	Unclassified	–
Bin_37	JBDIVZ000000000	3,376,157	686,036	99.5	0	19	14	20	475,147	Pseudomonadota	s__PRO5 sp003577275
Bin_38	–	324,866	14,842	3.51	0	3	102	5	3,068	Unclassified	–
Bin_39	–	229,895	55,875	0	0	–	17	28	61,044	Unclassified	–
**Bin_40**	**JBDIWA000000000**	**3,717,074**	**558,981**	**97.8**	**1.65**	**20**	**76**	**31**	**84,467**	**Planctomycetota**	**g__Kuenenia**
Bin_41	JBDIWB000000000	2,831,517	376,426	92.51	2.32	17	265	13	14,600	Pseudomonadota	g__Nitrosomonas
**Bin_42**	**JBDIWC000000000**	**4,159,250**	**617,649**	**97.8**	**2.2**	**20**	**115**	**25**	**63,828**	**Gemmatimonadota**	**g__SZUA-320**

^a^ High-quality MAGs are displayed in bold; low-quality MAGs do not have an accession number.

The data set enhances our understanding of deep underground microbial communities and facilitates the identification of functional genes with potential biotechnological applications.

## Data Availability

Accession numbers for raw sequence reads in GenBank National Center for Biotechnology Information (NCBI) are SRX24375474–SRX24375477. Accession numbers for metagenome-assembled genomes in GenBank NCBI are listed in Table 1.
